# Promoting professional and interprofessional relationship among nurses in Iran: a participatory action research

**DOI:** 10.25122/jml-2021-0047

**Published:** 2021

**Authors:** Hossain Keikha, Robabeh Memarian, Zohreh Vanaki

**Affiliations:** 1. Department of Nursing, Faculty of Medical Sciences, Tarbiat Modares University, Tehran, Iran

**Keywords:** nurse, professional relationship, interprofessional relationship, participatory action

## Abstract

Communication is the basis of nursing care and can have a significant effect on patient and community health. This study aimed to optimize the professional and interprofessional relationships of nurses. This participatory action research was conducted with 288 patients and 23 staff of one of the Reference Hospitals in Tehran, Iran, from 2019 to 2020. Interviews were simultaneously analyzed using the conventional content analysis method, and in the quantitative section, the communication skills checklist and a researcher-made questionnaire were used. Participants gave oral and written consent, and their confidentiality and anonymity were respected. Participants’ experiences showed that the implemented programs changed the work environment. The acquired basic concepts were improving the nurse’s responsibility and accountability, improving the quality of care and respecting the patient and the family. Quantitative data obtained before and after the implementation of change programs showed that patient satisfaction increased from 41.57±7.03 to 94.34±4.67 and patient and family training from 113.73±19.77 to 245.99±36.61. The length of hospital stay decreased from 5.52±2.47 to 3.57±2.35 days, nosocomial infections from 32.1±2.8 to 17.4±2.4 and readmission from 4.8±1.2 to 2.3±1.1. The results indicated that patients’ quality of care and safety was increased by optimizing the nurse’s professional and interprofessional relationship. Patients reported greater respect, and nurses had higher job satisfaction.

## Introduction

The ability to communicate effectively is one of the basic skills of social life [1]. In health professions, communication and communication skills have a pivotal role in satisfying employees and patients and solving their problems. Moreover, nursing is a profession where teamwork and communication are basic features [2]. Today, health systems seek to change attitudes from treatment superiority to care provision [3]. Likewise, nurses must be equipped with professional communication to provide proper care. It is very important for experts to believe that communication skills are the cornerstone of nursing practices [4]. Given the complexity of patient care and the importance of safe and high-quality care, having teamwork attitudes and professional communication skills are crucial [5–7].

Communication skills in nursing have two domains: communication with the treatment team and communication with the patient and family. Interprofessional communication refers to mutual respect for professional values, personal abilities, asking for opinion and consultation with the treatment team for decision-making [3], creating an equal opportunity for team members to share their knowledge and expertise in an environment enriched in mutual trust and respect, and interaction in the patient care. Due to the same work field of nurses and physicians and the mutual complementarity of these two professions, professional communication between them is inevitable compared to other treatment team members [8]. Research showed that effective communication between physicians and nurses could lead to positive outcomes such as increased information exchange, effective interventions, improved safety, improved staff morale, increased patient, and family satisfaction, and reduced readmission rate [9]. On the other hand, the study by Karanikola *et al.* showed that physician-nurses cooperation was very low, which is an important factor in increasing stress and burnout in nurses [10]. Numerous factors affect the lack of professional communication between physicians and nurses, including the level of familiarity with the job description and knowledge of effective communication [11, 12].

Factors affecting the type and quality of the nurse-patient professional communication include the nurse care method, the communication of the nurse with the patient and family, workplace atmosphere, and moral values [13], which have a great effect on caring behaviors and is the basis for providing high-quality care, promoting patients’ health, and providing their satisfaction [14, 15]. Research shows that the most common reason for patients’ dissatisfaction and confusion is the lack of effective communication and inconsistency between physicians and nurses in their behavior and speech [5]. As a result, the patient loses trust in the treatment team and reduces his cooperation because patients focus their relationship with the nurse on trust, and trust-building is the responsibility of the treatment staff, especially the nurse. Through professional communication with patients, nurses are aware of their care needs and try to protect them while establishing justice in providing appropriate care [16, 17]. It is of particular importance for elderly and illiterate patients.

Given the improved quality of care following the nurse and treatment members’ effective relationship with the patient, and patients’ dissatisfaction with the lack of participation in the treatment process, the empowerment of nurses and the effective professional communication is essential. However, the studies identified only focus on professional relationships and its contributing factors. Therefore, due to the lack of a practical program and literature in promoting professional relationships among nurses, the present study aimed to optimize professional relationships among nurses using the participatory action research method.

## Material and Methods

### Design

Participatory action research (PAR) was the most suitable method to optimize professional relationships among nurses, whose achievement depends on the teamwork of different professions. It is one of the best methods for problem-solving, where participants’ contribution to planning and implementation brings novel and creative models, and theoretical solutions are replaced with practical and logical ones [18–22]. In the present study, the Reason and Bradbury model, including four stages in PAR (i.e planning, action, observation, and reflection) that can be repeated in a cycle until objectives are achieved, was used [23].

### Study setting

The study was based on the researcher’s employment in the study environment. Observation of problems of care following the weakness of nurses in professional communication study was performed in the Department of Urology in Shohada-e-Tajrish Hospital in Tehran, Iran, a referring center for urinary tract disorders, between 2019 to 2020. The department included 28 active beds and admitted 3450 patients in the year before the study. The average number of admissions per month was 285, and the bed occupancy rate was 92%. This department admits patients referred from throughout the country.

### Sampling and data collection

Based on purposeful sampling, a total of 23 participants selected from the nursing team, including a nursing manager, an educational supervisor, a head nurse of the department of urology, 16 nurses, two physicians, and two faculty members, were enrolled in the study. Also, 12 urology medical assistants, three nurse assistants, three maids, and a secretary were selected. In addition, 19 females and 23 males, with a mean age of 41 years (ranged 26–56) and a mean work experience of 8.5 years (ranged 4–13), were included in the study. Also, patients referred to the department of urology as another group participating in the study before and after implementing optimization change programs were selected by a convenience sampling method, using the sample size formula. To estimate the maximum sample size, p=0.5 and d=0.05 were considered, and finally, the sample size was determined 288 selected from eligible patients.

To collect qualitative data, in-depth 30- to 45-minute interviews, focus group discussions, and field notes were utilized. This was done according to the availability and desire of the participants in the hospital environment and urology department. For quantitative data, to measure the performance of physicians and nurses and their interprofessional and professional relationship with patients and treatment team members, the communication skills checklist and a 53-item questionnaire, including demographic information and items on patient training, satisfaction, length of hospital stay, readmission, and nosocomial infection, were used. The expert panel was used to validate the content of the questionnaire, and its reliability was assessed by a test-retest with a correlation coefficient of 0.972.

### Procedures

The study was designed according to the basic problems extracted in 3 cycles. Furthermore, the study was implemented in 4 phases, starting in September 2017 and completed after 10 months.

#### Phase 1 (Initial reflecting & planning)

The first phase of the participatory action research was performed by collecting quantitative and qualitative baseline data. After obtaining informed consent, ensuring confidentiality, and explaining the study objectives, questionnaires were distributed to participants and collected after completion. The questionnaires were also completed by patients or their companions at discharge. Inclusion criteria were having physical and mental capabilities to answer and complete the questionnaire. Exclusion criteria were unwillingness to participate in the study. The researcher was present when the questionnaires were completed to answer questions of patients or their companions.

To collect the qualitative data, semi-structured interviews with open-ended questions (seven 30–45-minute sessions) were held. The recorded interviews were transcribed verbatim on 180 sheets, of which 115 problems were extracted, and after removing the duplicates, 35 remained. In the focus group discussions (two 60–90-minute sessions), gaps and opportunities were discussed by participants, and by integrating the problems, three main problems, including lack of nurses’ knowledge of various care methods and professional communication strategies, implementation of PAR, and nurses’ lack of knowledge, were determined and prioritized. After identifying the problems and the need for change, the solutions were evaluated. Then an action plan aimed at meeting nurses’ educational needs and modifying their professional communication was formulated. Resources, contributors, and actions were determined. In addition, a schedule was created, and executive decisions for the planned programs were made by holding two guiding sessions with hospital managers.

#### Phase 2 (Act)

Participants were involved in the implementation of action plans. Two one-day workshops were held for urology nurses on familiarity with care methods and effective communication. Then, nurses practiced patient-centered care techniques, especially the case method and interprofessional and professional relationships with patients and treatment team members. At the beginning of the shift, the head nurse assigned nurses to patients by case to plan care programs and supervised their performance. At the beginning of the shift, the nurse introduced herself to the patient and designed and implemented a care plan together, after examinations and evaluations. The patient shared his needs with the nurse and received the necessary medical services, care, and training. To strengthen the nurses’ behavior, the head nurse considered incentives, such as assigning advantages to a grade point and written appreciation. Moreover, based on the needs assessment, training workshops (one workshop per month) were held for nurses, which made them scientifically capable of implementing care programs and training the patient.

Bimonthly symposiums were held with nurses, urologists, and urology assistants. Job descriptions were reviewed for each, and existing problems caused by ineffective physician-nurse professional communication (patients’ complaints, complications caused by the lack of coordination between physicians and nurses etc) were discussed. It was decided to enhance the performance of the department of urology both qualitatively and quantitatively and reduce negative patient outcomes and complaints. It was also decided that all measures should be taken in a coordinated manner. The strengths and weaknesses of measures taken in the previous two months were reviewed in each session, and necessary amendments were made to the program.

#### Phase 3 (Observe)

The head nurse, clinical and educational supervisor, and the researcher regularly monitored the nurses’ performance in implementing the case care method, designing and implementing the care program, training the patient and family, and establishing effective communication. The nurse-patient communication, provision of nursing care, patient training, solving patients’ problems, and monitoring nurses’ performance in interprofessional communication skills were analyzed using a checklist.

After data collection, summarizing the observations, the study strengths and weaknesses found in the previous cycle, and corrective measures required for the next cycle were examined in the focus group discussions.

#### Phase 4 (reflection)

**Figure 1.**
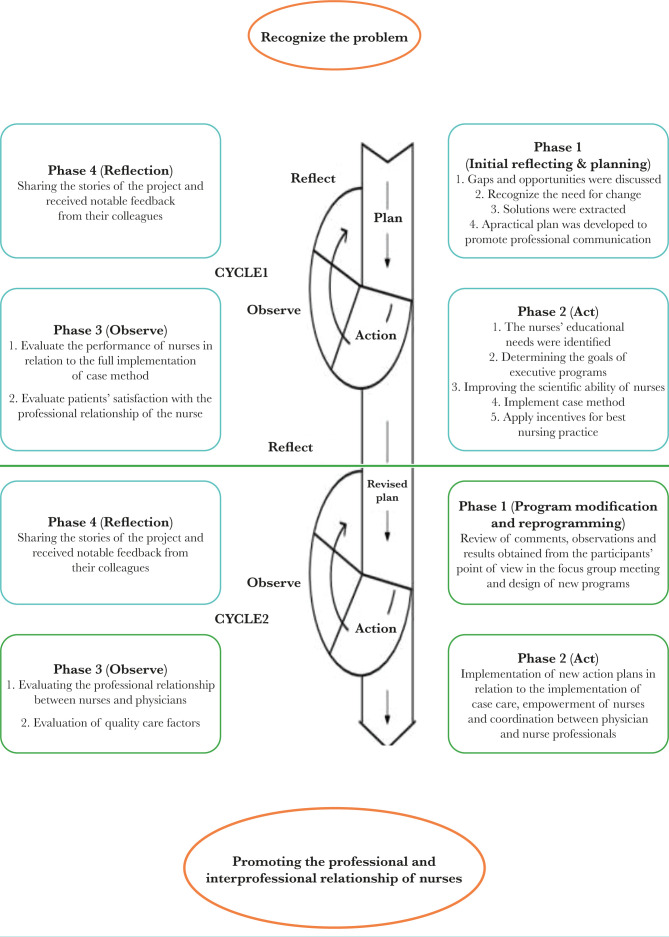
Schematic diagram of the stages of optimizing the professional relationship in nurses.

During the focus group meetings, participants shared their views, observations, and feelings, and reflected on the implementation of the program, and received feedback from colleagues. The meeting continued until reaching a consensus on each issue. Numerous reflection meetings were also held informally and consultatively with the participants and the research team during the implementation of the programs (Figure 1).

### Data analysis

Qualitative content analysis was used to reveal hidden meanings and patterns of qualitative data. By repeatedly listening to the audio files, the researcher transcribed the interviews verbatim and read them to achieve a general understanding. The transcriptions and identified semantic units were coded, and the semantic units were converted to the initial codes. Then, similar codes were classified in classes and subclasses to achieve meanings hidden in the data. Constant comparison technique and inductive approach were used to convert the initial data into basic knowledge [24]. Quantitative data obtained from the researcher-made questionnaire before and after the action research were examined using paired samples t-test and SPSS version 24 to determine the effect of measures taken to promote professional communication in nurses.

### Rigor

Given qualitative research challenges, validation criteria of qualitative research, including reliability, validity, generalizability, and verifiability, were used [25, 26]. Semi-structured interviews, field notes, reflections, and checklists contributed to the reliability of the data using the triangulation method. During the study, participants reviews, review of extracted data and codes made by the research team, accurate recording of research stages to enable auditing, and long-term engagement with participants and data were performed to increase data validity. Data generalizability was achieved through describing direct quotations, and to verify the data, two colleagues experienced in qualitative research reviewed data and documentation.

## Results

### Quantitative

Data from 288 patients referred to the department of urology showed that the level of education in most participants was below high school diploma (37.5%); the majority were male (89.5%) and married (85.4%), with a mean age of 52.60±6.60 years. The normal distribution of data was assessed using the Kolmogorov-Smirnov test. After comparing the data obtained before and after the participatory action research, the Chi-square test did not show a significant difference between the variables, and the participants were homogeneous in terms of demographic variables (Table 1). Regarding the care factors before and after promoting professional communication in nurses, the paired samples t-test showed a significant difference in patient satisfaction, training of patient and family, readmission rate, length of hospital stay, and prevalence of nosocomial infections (Table 2).

**Table 1.** Distribution of absolute and relative frequency of demographic variables in the studied patients.

**Variable**		**Before participatory action research**	**After participatory action research**	**The significance level**
**Age, yr**		**mean±SD**	**mean±SD**	
		51/43±6/80	52/60±6/60	P=0.126 t=1.55
		**frequency %**	**frequency %**	**P-value**
**Gender**	Male	279 (96.8)	258 (89.5)	x2=1.77 P=0.79
	Female	9 (3.2)	30 (10.5)	
**Marital status**	Married	280 (97.2)	246 (85.4)	x2=0.001 P=0.99
	Single	8 (2.8)	31 (10.7)	
	Widow(ed)	0	10 (3.5)	
	Divorced	0	1 (0.4)	
**Level of education**	Below diploma	134 (46.5)	108 (37.5)	x2=7.245 P=0.064
	Diploma	94 (32.6)	96 (33.3)	
	Student	(1.75)	6 (2.08)	
	Associate/bachelor’s degree	55 (19.15)	66 (22.9)	
	Higher education	0	12 (4.22)	

**Table 2.** Comparison of the scores of qualitative factors of care before and after the promotion of professional relationship in nurses.

**Care Factor**	**Before participatory action research, mean±SD**	**After participatory action research, mean±SD**	**P-value**
**Patients’ satisfaction**	41.57±7.03	94.34±4.67	0.0001
**Length of hospital stay**	5.52±2.47	3.57±2.35	0.001
**Nosocomial infections**	32.1±2.8	17.4±2.4	0.001
**Readmission**	4.8±1.2	2.3±1.1	0.029
**Training of patients and families**	113.73±19.77	245.99±36.61	0.001

### Qualitative

After implementing optimization programs, analysis of qualitative data consisted of 214 initial data, categorized in three sub-themes and one central theme. The experiences of the participants after participatory action research showed that the executive programs could change the work atmosphere, which was observed as “strengthened responsibility and accountability of nurses”, “improved quality of care”, and “respect for the patient and family”.

### Changes in the workplace atmosphere

Nurses’ perceptions and experiences showed that the workplace atmosphere was changed after participatory action research. In the new atmosphere, although the nurse worked in connection with the treatment team, a new pattern of professional and interprofessional relationship was formed, especially between the physician and the nurse, prioritizing the interests and rights of the patient. This increased the responsiveness and responsibility of nurses and physicians and improved their coordination and conformity. For example, the 57-year-old patient, after three days of hospitalization, said: “*I was admitted to different hospitals due to 11 years of bladder cancer but did not see anything like this department, in terms of attention, accuracy, and order. Doctors and nurses are very responsible, and I am very satisfied with my treatment process*”.

The physician of the department also added: “*I did not dare to entrust the patient to the nurse for a long time; I did it by myself, but now when I see that the nurses’ work mastery improved and she is even doing better and more accurately than me, I feel relaxed and leave the work to them*” (the 42-year-old urologist, three years of work experience).

### Responsibility and accountability

According to nurses, the implementation of the case care method could increase their responsibility and accountability. In what concerns the programs’ effect on the promotion of effective communication and implementation of the case care method in increasing the nurse’s responsibility and communication and training patients, the urologist nurse said: “*The case method has made the nurse to be more in contact with the patient and feel more responsible*” (a 40-year-old nurse with seven years of experience). Furthermore, the effect of this method of care on increasing the responsibility of nurses was such that it stirred the admiration of patients. A patient in this regard said: “*These nurses should be encouraged; they try to solve it when I have a problem and do not stop until resolving it*” (a 58-year-old patient diagnosed with an enlarged prostate).

### Improving the quality of care

According to the participants, the measures taken in participatory action research could improve the quality of care. For example, a nurse said: “*Scientific workshops and case methods improved patient care and increased their satisfaction*” (a 30-year-old nurse with five years of experience). The head nurse of the department of urology, referring to the effect of the measures taken to improve the quality of care, said: “*The quality of care has increased, from almost zero to a rather good state. Our nurses did not train at all before, but now they consider it a routine duty*”.

### Respect for the patient and family

According to participants, promoting professional and interprofessional relationships and prioritizing patients’ needs developed a sense of value and respect. One of the nurses highlighted the opportunity to spend more time communicating with patients, developing a sense of value and respect towards them, and increasing their satisfaction with care. She said: “*When my patients are determined, I have enough time to tell them about their problems and train them and see the satisfaction in the faces of patients and their families*” (a 26-year-old nurse with four years of work experience). A patient, pointing to the desired attention of nurses to patients, noted that: “*Whenever I have a question, they patiently explain it to me and have a special respect for my family and me*” (a 45-year-old patient diagnosed with urethral stenosis) and me.

## Discussion

Investigating participants’ experiences showed that empowering nurses and implementing case care methods brought a special place to nurses and led them to communicate with the patient and physician differently from the past. Furthermore, the nurse, as a professional, can independently design and implement patient care programs. This created trust between the treatment team and the patient, promoted self-care in patients, and led to a “change in the work atmosphere”. In the new work environment, care practices are patient-centered, and observance and respect for each other’s duties are designed and implemented by the physician and nurse. This increased the sense of responsibility and accountability in nurses, increased quality of care, improved value and respect for the patient and family, and brought a greater satisfaction in both the patient and the nurse. The workplace or organizational environment reflects the organizational values perceived by employees. Furthermore, it carries messages from the organizational environment shaping the employees’ perception of the goals and meaning of the organization, and subsequently, shaping their organizational behaviors [27–30]. Participants’ experiences showed that the atmosphere prevailing in the workplace significantly affects personal and organizational behaviors and interprofessional communications in the workplace. The establishment of case nursing improved the organizational climate and interprofessional communications, especially between nurses and physicians. In a study with a mixed-method design performed in a hospital in Brazil, Agreli *et al.* reported that improving organizational climate enhanced interprofessional communications [31]. Changing the workplace atmosphere from routine-centered to patient-centered and reducing nurses’ workload placed patients’ interests at the center of nursing philosophy. Based on the same philosophy, nurses in the present study had effective communications with patients and considered patient training and empowering self-care their professional duty. Bachnick *et al.* also reported a significant relationship between patient-centered care and the workplace atmosphere of the nurse. Patients felt they received better care and more information in such an environment [32], which aligns with the present study findings.

One of the findings of this study was increased responsibility and accountability in nurses. Participants’ experiences showed that the symposiums and establishment of the case care method improved the communication and coordination between nurses and physicians, reduced their controversies, and accelerated the treatment and care procedures. According to Iliadi *et al.*, interprofessional communication and cooperation and feeling responsible and accountable are closely correlated and are essential features of being a professional [33]. Furthermore, Russell *et al.* found that while functional nursing interrupts care, reduces nurse-patient communication, and decreases the sense of responsibility and accountability in nurses, patient-centered nursing techniques, such as case care, increase their responsibility and accountability [34].

The present study results also indicate that improving quality of care and promoting respect for the patient and family are two other outcomes of promoting professional and interprofessional relationships in nurses. Numerous studies reported patient training [35], length of hospital stay and complications [36], patient satisfaction [37], and readmission rate as examples of the quality of care. The results of the present study showed that after the implementation of change programs, these variables improved and showed a significant difference, indicating an improvement in the quality of care (Table 2). In this regard, the results of the study by Cui, X. *et al.* showed that effective communication with the patient had a direct relationship with the quality of care, and by improving it, the quality of care is improved [38]. From the participants’ point of view, improving the relationship with the patient increased the time spent with them, and their training caused a sense of value and respect for them. After implementing participatory action research, a sustainable behavior change was achieved in establishing professional and interprofessional relationships in nurses, which was due to the participation of nurses in decision-making, planning, implementation, and evaluation stages. Therefore, it is suggested to use the study method to answer some of the health system problems.

## Conclusion

The findings of the study showed that the scientific capability of nurses and the establishment of case care methods caused changes in the work atmosphere and improved the professional communication of nurses with patients and interprofessional relationships with physicians. An effective factor in promoting professional communication that led to the acceptance and implementation of changes made after action research was the participation of nurses in decision-making and planning throughout the research. As a result of these changes, the quality of care and patients’ safety increased; patients felt more value and respect, and nurses had more job satisfaction.

## Acknowledgments

### Conflict of interest

The authors declared that there is no conflict of interest.

### Ethics Approval

The article was created from the Ph.D. dissertation in nursing. The research project protocol was approved by the Ethics Committee of Tarbiat Modares University (ethical code: IR.TMU.REC.1396.619).

### Consent to participate

The confidentiality of information and freedom of the participants to withdraw from the study at any stage were observed in the research. The study objectives and voluntary nature of participation were explained to subjects, and they were assured of the information, anonymity and freedom to withdraw from the study at any stage. All information obtained through observation, interviews, sessions, and the recording of sessions with patients and their companions, were kept confidential.

### Personal thanks

The authors acknowledge their gratitude to the head of the hospital, the nursing managers, the head nurse, and the nurses of the Department of Urology in Shohada-e-Tajrish Hospital for their cooperation with the study.

### Authorship

HK, RM and ZV contributed to writing the original draft, methodology. HK, RM contributed to conceptualizing HK, ZV contributed to data collection.

## References

[1] Mohammadi S, Nakhaei N, Borhani F, Roshanzadeh M (2013). Moral intelligence in nursing: a cross-sectional study in East of Iran.. Iranian Journal of Medical Ethics and History of Medicine.

[2] Amini K, Negarandeh R, Ramezani-Badr F, Moosaeifard M, Fallah R (2015). Nurses’ autonomy level in teaching hospitals and its relationship with the underlying factors.. International journal of nursing practice.

[3] Mehrabi M, Madanipour A, Ahmadnia S (2016). The sociological study of nurse-physician professional relationship in Iran.. Iranian journal of nursing and midwifery research.

[4] H AL (2011). Experience professional association of nurses in educational hospitals:a phenomenological study.. J Mazand Univ Med.

[5] Mahdizadeh M, Heydari A, Moonaghi HK (2015). A Review of the Clinical Interdisciplinary Collaboration among Nurses and Physicians.. Open Journal of Nursing.

[6] Georgiou E, Papathanassoglou ED, Pavlakis A (2017). Nurse-physician collaboration and associations with perceived autonomy in Cypriot critical care nurses.. Nursing in critical care.

[7] Tebyanian H, Karami A, Motavallian E, Aslani J, Samadikuchaksaraei A, Arjmand B, Nourani MR (2017). Histologic analyses of different concentrations of TritonX-100 and Sodium dodecyl sulfate detergent in lung decellularization.. Cell Mol Biol (Noisy-le-grand).

[8] Hughes RG (2008). Patient Safety and Quality: An Evidence-Based Handbook for Nurses..

[9] Matziou V, Vlahioti E, Perdikaris P, Matziou T, Megapanou E, Petsios K (2014). Physician and nursing perceptions concerning interprofessional communication and collaboration.. Journal of interprofessional care.

[10] Karanikola MN, Albarran JW, Drigo E, Giannakopoulou M, Kalafati M, Mpouzika M, Tsiaousis GZ, Papathanassoglou ED (2014). Moral distress, autonomy and nurse-physician collaboration among intensive care unit nurses in Italy.. J Nurs Manag.

[11] Motamed-Jahromi M, Jalali T, Eshghi F, Zaher H, Dehghani L (2015). Evaluation of professional autonomy and the association with individual factors among nurses in the Southeast of Iran.. Journal of Nursing and Midwifery Sciences.

[12] Sharifiyana M, Zoharianbohi S, Abadiuthor N, Dabirian A, Alavi Maj H. (2016). Evaluation of participation in clinical decision making by nurses in selected hospitals of shahid beheshti university of medical sciences.. The Journal of Urmia Nursing and Midwifery Faculty.

[13] Sharafi S, Chamanzari H, Bazi A, Mazloom SR, Maghsoodi S, Rajabpour M (2015). Impact of nursing care delivery system “case method and primary nursing” on nurse-patient interaction in CCU.. Q J Nurs Manage.

[14] Sharafi S, Chamanzari H, Pouresmail Z, Rajabpour M, Bazzi A (2018). The Effect of Case Method and Primary Nursing Method on the Social Dimension in Quality of Patient Care.. Journal of Holistic Nursing and Midwifery. [Research]..

[15] Lotfi M, Zamanzadeh V, Valizadeh L, Khajehgoodari M (2019). Assessment of nurse–patient communication and patient satisfaction from nursing care.. Nursing open.

[16] Phillips G (2016). Nurses are best placed to ensure the ethical application of DNRs.. Nursing Standard (2014+).

[17] Fahlberg B, Foronda C, Baptiste D (2016). Cultural humility: The key to patient/family partnerships for making difficult decisions.. Nursing2019.

[18] Danley K, Ellison M (1999). A Handbook for Participatory Action Researchers.. Implementation Science and Practice Advances Research Center Publications.

[19] Mosaddad SA, Beigi K, Doroodizadeh T, Haghnegahdar M, Golfeshan F, Ranjbar R, Tebyanian H (2021). Therapeutic applications of herbal/synthetic/bio-drug in oral cancer: An update.. Eur J Pharmacol.

[20] Yazdanian M, Tabesh H, Houshmand B, Tebyanian H, Soufdoost RS, Tahmasebi E (2020). Fabrication and properties of βTCP/Zeolite/Gelatin scaffold as developed scaffold in bone regeneration: in vitro and in vivo studies.. Biocybern Biomed Eng.

[21] Yazdanian M, Rahmani A, Tahmasebi E, Tebyanian H, Yazdanian A, Mosaddad SA (2021). Current and advanced nanomaterials in dentistry as regeneration agents: an update.. Mini-Rev Med Chem.

[22] Mosaddad SA, Yazdanian M, Tebyanian H, Tahmasebi E, Yazdanian A, Seifalian A, Tavakolizadeh M (2020). Fabrication and properties of developed collagen/strontium-doped Bioglass scaffolds for bone tissue engineering.. J Mater Res Technol.

[23] Reason P, Bradbury H (2001). Handbook of Action Research: Participative Inquiry and Practice; Sage.

[24] Coughlan P, Coghlan D (2002). Action research for operations management.. International journal of operations & production management.

[25] Polit DF, Beck CT (2008). Nursing research: Generating and assessing evidence for nursing practice.

[26] Tebyanian H, Karami A, Motavallian E (2017). A Comparative Study of Rat Lung Decellularization by Chemical Detergents for Lung Tissue Engineering.. Open Access Maced J Med Sci.

[27] Ghlaeei A, Mohajeran B, Divband A (2017). Investigating the Effect of Organizational Climate on Organizational Citizenship Behavior of Teachers with Role of Mediate Individual Accountability.. J School Admin.

[28] Moghadam ET, Yazdanian M, Tahmasebi E, Tebyanian H, Ranjbar R, Yazdanian A, Seifalian A, Tafazoli A (2020). Current herbal medicine as an alternative treatment in dentistry: In vitro, in vivo and clinical studies.. Eur J Pharmacol..

[29] Tebyanian H, Karami A, Motavallian E, Samadikuchaksaraei A, Arjmand B, Nourani MR (2019). Rat lung decellularization using chemical detergents for lung tissue engineering.. Biotech Histochem..

[30] Soufdoost RS, Yazdanian M, Tahmasebi E, Yazdanian A, Tebyanian H, Karami A, Nourani M.R., Panahy Y (2019). In vitro and in vivo evaluation of novel Tadalafil/β-TCP/Collagen scaffold for bone regeneration: A rabbit critical-size calvarial defect study.. Biocybern Biomed Eng.

[31] Agreli HF, Peduzzi M, Bailey C (2017). The relationship between team climate and interprofessional collaboration: Preliminary results of a mixed methods study.. Journal of Interprofessional Care.

[32] Bachnick S, Ausserhofer D, Baernholdt M, Simon M (2018). Patient-centered care, nurse work environment and implicit rationing of nursing care in Swiss acute care hospitals: A cross-sectional multi-center study.. International Journal of Nursing Studies.

[33] Iliadi P (2010). Accountability and Collaborative Care: How Interprofessional Education Promotes.. Health Science Journal.

[34] Roussel L, Swansburg RC, Swansburg RJ Management and Leadership for Nurse Administrators.

[35] Gröndahl W, Muurinen H, Katajisto J, Suhonen R, Leino-Kilpi H (2019). Perceived quality of nursing care and patient education: a cross-sectional study of hospitalised surgical patients in Finland.. BMJ Open.

[36] Brasel KJ (2007). Length of Stay.. Archives of Surgery.

[37] Al-Abri R, Al-Balushi A (2014). Patient Satisfaction Survey as a Tool Towards Quality Improvement.. Oman Medical Journal.

[38] Cui X, Zhou X, Ma LL, Sun TW, Bishop L, Gardiner FW, Wang L (2019). A nurse-led structured education program improves self-management skills and reduces hospital readmissions in patients with chronic heart failure: a randomized and controlled trial in China.. Rural Remote Health.

